# Radon Exposure—Therapeutic Effect and Cancer Risk

**DOI:** 10.3390/ijms22010316

**Published:** 2020-12-30

**Authors:** Andreas Maier, Julia Wiedemann, Felicitas Rapp, Franziska Papenfuß, Franz Rödel, Stephanie Hehlgans, Udo S. Gaipl, Gerhard Kraft, Claudia Fournier, Benjamin Frey

**Affiliations:** 1 Biophysics Department, GSI Helmholtzzentrum für Schwerionenforschung GmbH, 64291 Darmstadt, Germany; a.maier@gsi.de (A.M.); j.wiedemann@gsi.de (J.W.); f.rapp@gsi.de (F.R.); f.papenfuss@gsi.de (F.P.); g.kraft@gsi.de (G.K.); c.fournier@gsi.de (C.F.); 2 Department of Radiotherapy and Oncology, University Hospital Frankfurt, Goethe-Universität Frankfurt am Main, 60590 Frankfurt am Main, Germany; franz.roedel@kgu.de (F.R.); stephanie.hehlgans@kgu.de (S.H.); 3 Translational Radiation Biology, Department of Radiation Oncology, Universitätsklinikum Erlangen, Friedrich-Alexander-Universität Erlangen-Nürnberg, 91054 Erlangen, Germany; udo.gaipl@uk-erlangen.de

**Keywords:** radon therapy, low doses, α-particles, clinical studies, anti-inflammatory effects, changes immune activation, osteoimmunological changes

## Abstract

Largely unnoticed, all life on earth is constantly exposed to low levels of ionizing radiation. Radon, an imperceptible natural occurring radioactive noble gas, contributes as the largest single fraction to radiation exposure from natural sources. For that reason, radon represents a major issue for radiation protection. Nevertheless, radon is also applied for the therapy of inflammatory and degenerative diseases in galleries and spas to many thousand patients a year. In either case, chronic environmental exposure or therapy, the effect of radon on the organism exposed is still under investigation at all levels of interaction. This includes the physical stage of diffusion and energy deposition by radioactive decay of radon and its progeny and the biological stage of initiating and propagating a physiologic response or inducing cancer after chronic exposure. The purpose of this manuscript is to comprehensively review the current knowledge of radon and its progeny on physical background, associated cancer risk and potential therapeutic effects.

## 1. Introduction

Radon is a naturally occurring, radioactive noble gas that contributes as the largest single fraction to radiation exposure from natural sources [1]. It is produced by various decay chains of uranium and thorium and has no stable isotopes [2]. However, there are three naturally occurring isotopes: ^222^Rn with a half-life of 3.825 days, originating from the uranium series, ^220^Rn (thoron, T1/2 = 55.6 s) derived from the thorium series and ^219^Rn (actinon, T1/2 = 3.96 s) from the actinium series [3]. As these isotopes are noble gases, there are no known chemical interactions at physiological temperatures [4].

In 1899, Rutherford and Owens discovered radiation emanating from thorium oxide and uranium [5]. In further studies, Rutherford identified a radioactive substance, permanently emitted from thorium compounds, which turned out to be ^220^Rn [6]. In parallel, Marie and Pierre Curie discovered the ^222^Rn isotope by studying the emanation from radium, which stayed radioactive for several days due to the comparatively long half-life of this isotope [7]. Based on the work of Rutherford and Curie, Dorn confirmed their results with both uranium and thorium [8], while Debierne discovered the isotope ^219^Rn by measuring radioactive emanation from actinium [9].

Due to their half-lives of 3.8 days and 55.6 s, respectively, ^222^Rn and ^220^Rn isotopes are the only radon-nuclides that exist long enough to emanate from natural rocks and soil where they are formed. Due to its short half-life, ^220^Rn has a shorter diffusion length than ^222^Rn. Nevertheless, if ^220^Rn is present, it can contribute significantly to the total inhalation dose and should not be neglected [10]. Thus, both isotopes, ^222^Rn and ^220^Rn, are the only significant contributors to human radon exposure from natural sources [1]. After emanation in ambient air, radon isotopes accumulate indoors and represent the most important contributor to annual radiation dose of the population [11,12]. However, the radon activity concentrations in homes highly depend on geological conditions such as the uranium versus thorium content and the gas permeability of the soil. In addition, anthropogenic factors such as building materials, ventilation systems, or living habits play a significant role. Interestingly, some building materials are not only sources for indoor ^222^Rn but also ^220^Rn exposure [1], and its concentration varies considerably with the distance from the wall and the airflow [13]. All these facts together lead to large regional differences [12,14,15] and, in average, to higher radon concentration indoors than outdoors [16]. Regions like Kerala in India and cities like Yangjiang (China) or Ramsar (Iran) have particularly high radon concentrations in soil and indoors [17]. However, not only indoor accumulation, but also showering with radon-containing water releases radon to moist air, which represents a substantial source of radon exposure [18]. This fact is supported by measurements of the radon activity concentration in spa treatment rooms during filling of the bathing tubes, enhancing radon activity concentrations [19]. Nevertheless, the level of radon daughter nuclides usually remains low during filling, since they attach to vapor and are removed by ventilation and air circulation [20]. Intake of radon via drinking radon-containing water represents a minor source of exposure compared to inhalation [21].

Both radon isotopes disintegrate into several unstable daughter nuclides, emitting different radiation types (see Table 1). 

After decay in air, the nuclides react in less than one second with trace gases and air vapor, forming clusters of 0.5–5 nm size, also called the “unattached progeny”, which are positively charged and highly mobile [23,24]. Within 100 s, those clusters may attach to aerosol particles by diffusion, described by gas kinetic laws. The parameter that mostly influences the fraction of attached daughter nuclides is the number of aerosols [25] with the influence of electrostatic forces considered to be negligible [23,26]. The formed particles build the “attached progeny” for which diffusion coefficient measurements showed three distinct size ranges. These are called nucleation mode covering sizes from 10–100 nm, accumulation mode with particle sizes ranging from 100–450 nm and the coarse mode for particles larger than 1 µm [1]. The size distribution is strongly influenced by the aerosol mixture in the air. Accordingly, all studies show slightly different results but were consistent in the fact that the highest activity originates from radon decay products bound to aerosols associated with the accumulation mode [1,25,27]. Moreover, measurements showed that over 90% of the activity is associated with the “attached progeny” while the “unattached progeny” accounts for only 10% [21,23], being, in turn, three to five times more effective in dose commitment due to its smaller size [28].

Once built, solid daughter nuclei deposit on surfaces such as walls and furniture by different mechanisms (sedimentation, impaction, interception and diffusion), resulting in a lower activity concentration of the decay products in indoor-air than expected from equilibrium with radon [23,27]. This and other removal processes reduce the concentration of radon decay products, depending on a number of interlinked parameters such as the loss by radioactive decay, ventilation or the aforementioned deposition on room surfaces [29].

## 2. Intake and Distribution of Radon in the Human Organism

There are different routes of intake for radon and its solid progeny into the human body: during inhalation through the epithelial surfaces of lung, uptake through the skin while bathing in radon-containing water and by ingestion via the gastrointestinal tract by drinking radon-containing water. The incorporation of radon via drinking water is not further addressed here, as this route only plays a minor role for therapeutic application as well as public health [21].

### 2.1. Inhalation

The primary route of incorporation is inhalation, which occurs in radon galleries and in radon-contaminated buildings, leading to diffusion of radon gas through the lung epithelium and deposition of the solid progeny in the lung. The α-particles originating in this decay chain are the major contributors to the physical absorbed dose, whereas β- and γ-decay contributes to around 10% of the deposited energy [28,30]. For radiation protection purposes, a proper risk assessment is necessary and the exposure to certain radon activity concentrations has to be converted into an effective dose. For this, the absorbed energy has to be determined leading to the physical dose which is multiplied with radiation and organ specific weighting factors, considering the ionization pattern of various radiation types and the relative sensitivity of different tissues.

Considering the inhaled progeny, the lung equivalent-dose contributes to more than 95% of the total effective dose [31] because the progeny will largely deposit on the surface of the respiratory tract and decay before clearance can occur [1,28]. Additionally, simulations with various models of chronic exposure suggest that decay products cover more than 95% of the total effective dose received by exposure to radon while the radon gas itself contributes to less than 5% [1,28,31,32,33]. The reason is that, presumably, only about 1% of the inhaled gas is absorbed by the blood [33,34]. Assuming the inhalation of pure radon gas without progeny, simulations revealed that 30–50% of the effective dose is deposited in the lung due to radon decay in the airways.

**Figure 1:**
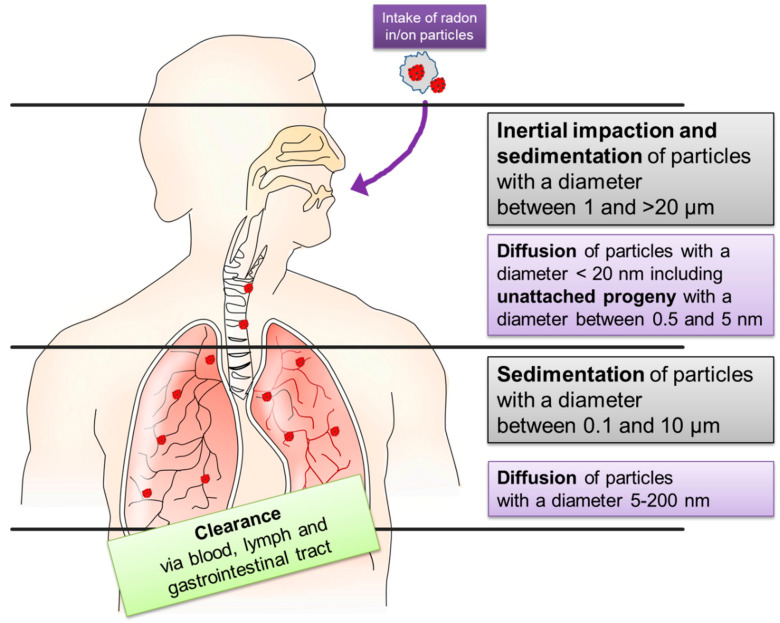
Different deposition mechanism for attached and unattached particles with various particle sizes. Drawing was taken from OpenClipart-Vectors on Pixabay under Creative CommonsCC0.

Model calculations based on animal experiments describe the deposition of particles, which is different for attached and unattached radon progeny. The aerosols to which the progeny are attached show different characteristics (size, shape, and others). If combined with detailed morphometry and physiological parameters of the lung (breathing pattern and lung geometry) three different deposition mechanisms are to be discriminated: inertial impaction, sedimentation, and diffusion (see Figure 1). Despite these measurements not being performed with radon decay products, the basic mechanisms are supposed to be the same. Although there are a lot of simulations, the exact dose to different parts of the lung remains unclear as there are no experimental data to ascertain these simulations.

In the upper region of the respiratory tract (nasopharynx, trachea, and upper bronchi), particles with 2–20 µm diameter keep their trajectory, despite changes in direction of air stream because of their inertial momentum and get stuck there. This process is called inertial impaction. Sedimentation describes the settling of smaller particles (0.1–50 µm) due to gravity in the upper respiratory tract and mainly in bronchioles and alveoli. Diffusion due to Brownian motion increases with decreasing particle size (<0.2 µm) and predominates in the gas exchange regions of the lung, whereby the “unattached progeny” (0.5–5 nm) mainly deposits directly after entering the respiratory tract (see Figure 1). The total lung deposition shows a minimum for particle sizes ranging from 0.2–0.5 µm [35,36,37] as these particles are too lightweight for sedimentation but have a decreased diffusion coefficient due to their size. Moreover, turbulences and inverse flows cause an inhomogeneous deposition pattern and hot spots of deposition at bifurcations from larger into smaller airways [37].

In the lung models used by the International Commission for Radiation Protection (ICRP), are considered to have different sensitivities to radiation at different regions of the respiratory tract [1], and basal and mucous cells in the bronchial epithelium are regarded as particularly radiosensitive [38]. Further, simulation studies suggest that the highest dose from radon decay products is deposited at the bifurcation of the trachea [39], with the latter not to be the most sensitive region.

Besides deposition, the reversed process of removal has to be considered for dose estimation. After deposition, solid daughter nuclei can be eliminated by clearance mechanisms. General knowledge of the physiological mechanisms suggest three primary routes of clearance: via the bloodstream, the lymphatic drainage system, or the gastrointestinal tract [35], depending on the characteristics of the particles used and the settings of the respective experiments (e.g., particle number, location in the respiratory tract) [40,41,42]. In the trachea or bronchial tubes, clearance mostly occurs by mucociliary transport or phagocytosis by pulmonary alveolar macrophages. Below the ciliated airways in the area of gas exchange, clearance, and transport to other tissues takes place via the bloodstream, lymphatic channels, or phagocytosis. Depending on the main mechanism of clearance different regions of the respiratory tract show different predominant clearance rates, whereby a superimposition of different clearing rates can occur in one lung region [35,40,43].

### 2.2. Incorporation via Skin

In homes and especially in radon galleries, inhalation of radon via the lung plays a dominant role in radon uptake. In spa treatments with radon-containing water and in vapor cabinets, radon and its progeny enter the body via the skin epithelium, while inhalation only plays a minor role as the head of the patients usually remains outside the treatment tub in the well-ventilated treatment rooms [44]. In open bath tubes radon- and progeny-containing vapor is also inhaled through the lung [45].

As for the lung epithelium, radon can diffuse through the skin. When reaching the bloodstream, it is distributed throughout the body. A part is transported back to the lung and exhaled [46]. After entering radon-containing water, the radon activity concentration in the exhalation air of patients undergoing spa therapy increases very fast, reaching saturation levels after approximately 20 min [46]. Afterwards, it is reported that radon is removed via breathing in an exponential fashion within a few minutes [44], whereas the decay products are mainly eliminated via excretion [47]. This means that the uptake and elimination of radon in and out of the human body is a fast process, while the decay products can stay in the body for a considerably longer time.

For radon bathing, it was stated that a minor fraction of the radon progeny will be adsorbed by the skin, but the major part will be desorbed after their decay. In radon galleries and vapor bathes, this is not the case, and radon progenies will stay on the skin. In both cases, radon progeny deposit a considerable energy to the skin, which is higher after treatment in a radon gallery than after radon bath [48,49]. According to experimental results reported by Tempfer and colleagues, the radon progeny activity shows an exponential decrease with skin depth to 20–30% of the surface activity at a depth of 20 µm [48]. This is attributed to diffusion and transport of progeny along hair capillaries and micro-crevices [48].

### 2.3. Distribution

Measurements of the distribution of primary radon in the human body after exposure are scarce. Inhalation experiments with the radioactive noble gas krypton show that the uptake and elimination of krypton (^79^Kr, ^81^Kr) activity at knee and arms was influenced by the rate of blood flow, as better circulation leads to faster kinetics with half times between 6–320 min [50]. One of the few measurements of radon activity concentrations in humans was obtained by exposure of a test person to high levels of radon and subsequent analyses of the radon concentration in the exhaled air. Five distinct elimination coefficients were determined, which were correlated with different body sites to conclude on the retention and exhalation of radon gas due to its solubility in body tissues [51]. There are few additional data mainly used for modelling purposes on the retention of radioxenon in the human body [52], in dogs [53], and for krypton in guinea pigs [54].

Most of the data for radon solubility are derived from animal experiments obtained in rats, where the highest value was determined for adipose tissue (omental fat), with a more than 10 times increased solubility as compared to other tissues like brain, liver, or muscle [34,38,55], although the maximum radon concentration is attained much slower. Adipose tissue shows a two-component built-up with different time constants of 21 and 138 min [56]. Calculations further indicate an elevated dose to red bone marrow due to the high fat cell content [57]. More recently, comparable results for the solubility of radon in different organs were obtained in mice [55]. In vitro measurements of radon solubility coefficients in fatty acids indicate an interrelationship between the number of carbon atoms in the fatty acid and the solubility per molecule [58]. In addition, radon is not equally distributed between different compounds. Although radon solubility is highly dependent on external conditions like temperature or salinity when measuring in water [59], measurements and molecular dynamic simulations revealed that radon is more soluble in fatty acids than in water because of the stronger cross bonding of the water molecules compared to fat [60].

In contrast to the pure solubility, which is a passive process, radon, in addition, is transported actively via the bloodstream and its further exchange via diffusion is governed by radon solubility. The resulting inhomogeneous distribution between different tissues determines the dose to different organs. For subsequent dose calculations, measurements of activity concentrations, and determination of diffusion and solubility of radon in different tissues is required [28,61].

For such multiparametric calculations, model systems are used, which usually consist of different compartments with specific morphometric and physiological parameters, conterminously with different tissue and organs in the human body [31,62,63]. Even though a model for the calculation of absorbed dose rates to organs and tissues in mice, rats, and humans, provide similar values for the different species [34], the input parameters for radon distribution in these models usually are derived from animal data, making it difficult to transmit these values to humans.

Besides the dependence on the model and the physiological parameters, the calculated doses are highly dependent on factors like exposure duration, radon activity concentration, amount of radon decay products in air, and size of the formed particles [64]. Therefore, we consider it difficult to provide exact dose values, but some statements on the relative dose depositions seem to be supported by the data. The highest dose is deposited in the lung, mainly caused by radon decay products during inhalation of primary radon [28]. This is supported by biodosimetric measurements in mice after radon exposure, which show a three times higher dose in the lung compared to kidney, heart, or liver [65].

As the reported measurements and simulations are consistent regarding high solubility of radon in adipose tissue, it seems reasonable to assume that this is also the reason for the calculated higher doses in bone marrow and female breast, which is approximately half of the dose to the lung [31,62]. However, the inner organs outside the respiratory tract receiving the highest dose from radon decay products are the kidneys [28].

In conclusion, the question remains whether this inhomogeneous distribution and the hot spots in fatty tissue are important to clarify the mechanistic basis in the clinical effects observed in patients and must be related to the exposure, i.e., the integrated radon activity concentrations. Nevertheless, radon solubility coefficients are weak points in these models, as these values strongly depend on the scarce parameters as mentioned before [31,34,63].

## 3. Cancer Risk

Risk estimation is important for chronic exposure to radon at working places as well as in homes, but it is indispensable for a balanced risk to benefit evaluation for therapeutic applications. The epidemiological studies that are available center on chronic (i.e., years of daily, continuous) exposure, either occupational or environmental. In contrast, non-chronic radon therapy typically covers up to 10 treatment sessions (i.e., treatment time of 20 to 60 min daily) in one series and normally performed once a year. Unfortunately, there are no epidemiologic data about a therapeutic exposure to radon reported up to now.

The short-living, α-emitting decay products together with the primary radon contribute significantly to the exposure of humans from natural sources [66]. For a long time, there has been strong evidence that these isotopes are the causative agent for lung cancer induction in miners when deposited in the respiratory tract. So, an increased risk for the development of lung cancer was shown for occupational exposure of minors in mountain galleries to radon and its progeny [16,67]. There is consent that environmental exposure to radon is the second leading cause of lung cancer induction after cigarette smoking [14,17]. The excess risk for lung cancer induction due to radon exposure and due to smoking act synergistically in a sub-multiplicative interaction, while an additive relation was rejected by modeling the epidemiologic data sets [68,69].

For risk estimation, the evaluated occurrence of lung cancer must be related to the exposure, i.e., the integrated radon activity concentrations. One problem for epidemiological studies is that the radon activity concentrations during exposure to radon and its progeny depend on environmental and behavioral factors, leading to highly variable concentrations. The exact determination would be important for risk assessment but is difficult to achieve, in particular retrospectively [67]. However, epidemiological studies for chronic exposure show a significant increase for risk of lung cancer with increasing radon concentrations [70,71] and exposure duration [72]. In the study of Darby et al., an increase in the risk of lung cancer of 16% per 100 Bq/m3 (95% confidence interval 5–31%) was found in a collaborative meta-analysis of 13 case-controlled studies [73]. These findings and comply with cohort studies of miners with low exposure rates over long times [74]. Age at and time since exposure modifies the excess relative risk per cumulative exposure. The risk decreases significantly by increasing the time at and since exposure [68]. Overall, lung cancer mortality and radon exposure are correlated linearly [74] without threshold [73]. When adjusting the absolute lifetime risk of lung cancer for smoking status, the risk for never smokers is much lower than that for smokers [72]. The conclusion of these epidemiologic studies is that radon represents a significant public health problem [75], when chronic exposure takes place.

During radon therapy (indications are listed in Table 2), the doses received by the patients in the course of one treatment series (typically consisting of ten sessions of one hour each) are in the same order of magnitude as for the natural annual background radiation due to radon. The major difference is the much shorter time period in which the patients obtain this dose and consequently, the higher dose rate. Therefore, the risk of a radiation/radon-induced severe effects of a radon treatment as prescribed by physicists is only fragmentarily described. The best description of side effects is from Franke A and Franke T analyzing the data of the so called IMuRa trial [76]. They described no acute side effects, which exceed a minor degree and they do not report any radiation-induced severe side effects, even at long term observations. These reports correspond with any other description of trials dealing with radon treatment, as summarized in Table 3. Today, there are two major concerns when extrapolating the carcinogenic effects on patients treated with radon bathes or gallery visits. On the one hand, the dose and duration as well as the frequency of radon contact (including inhalation and skin contact) is completely different. On the other hand, the patients are consuming or have consumed pain relieving drugs for years. The exclusion of the side effects from the radon-induced ones at short or even long follow up time is nearly impossible. Therefore, it is difficult to specify an additional risk due to radon therapy, as there are again additionally many unknown factors like natural background in patient homes due to radon or smoking behavior. Additionally, the impact of dose rate lowers the transferability of risk estimates related to the different exposure scenarios [77]. Precisely for this reason, a reliable value for the excess risk of radon therapy by radon itself cannot be calculated from retrospective or epidemiologic data. So, a potential risk from radon faces the described effect of pain relief even for long term and is therefore ethically negligible.

Besides induction of lung cancer, other organs could be affected. For instance, there are studies on the effects by plate out of radon progeny on the skin to investigate ulceration and dermal atrophy as potential effects. These non-cancer effects were considered as unlikely to occur for irradiation by those nuclides, as they require an irradiation of the dermis. During exposure, deeper layers that cannot be reached by these α-particles would need to be irradiated, and this makes a correlation between radon progeny exposure and skin cancer induction unlikely [78]. However, an excess risk of basal cell carcinoma was found for residents of geothermal areas in Iceland chronically exposed to elevated levels of radon, but confounding factors could also not be excluded [79]. The results of a Danish radon study with 51,445 subjects and a mean follow-up of 13.6 years suggests a potential effect on the development of basal cell carcinoma, but again confounding factors like sunlight could not be excluded [80], making the statements on skin effects of radon less reliable.

There is some evidence for a correlation between chronic exposure to radon and mortality due to malignant brain tumors. Nevertheless, this study had a non-robust epidemiological design to confirm this finding [81]. Additionally, in studies on the occurrence of the radon decay products ^210^Po and ^210^Bi in the brain of persons suffering from Alzheimer’s or Parkinson’s disease, an inhomogeneous distribution of these nuclides was found, but these findings are not sufficient to draw conclusions concerning correlative underlying mechanisms [82,83].

Suggestions were made on a correlation between myeloid leukemia and chronic radon exposure [57], and a significant positive association between indoor radon and acute myeloid leukemia incidence in children was observed [84]. In sum, based on these epidemiologic data, ^222^Rn and its decay products are classified as carcinogenic to humans for lung cancer by the International Agency for Research on Cancer (IARC), while data are inconclusive for other cancer entities [85]. In addition, a latency time between irradiation and development of malignancies of 5–7 years for leukemia and 10–60 years for solid tumors was observed [86]. Additionally, the age at exposure and the time since exposure seem to play a role for the risk due to irradiation. This makes it difficult to estimate the cancer risk after therapeutic application of radon.

## 4. Radon as a Therapeutic Agent

In spite of the aforementioned risk associated with radon exposure, it is used as a therapeutic agent. In ancient history, applications of “hot bathes” as well as inhalation were basic medical principles applied for treatment of inflammatory diseases. At the beginning of the 20th century radon was found to be a therapeutic agent in several thermal springs [87,88]. Therefore, the rise of so-called radon spas started and the application of radon for relief of pain caused by chronic degenerative diseases became popular. Although there was only clinical experience, the results of several recent trials suggest a positive effect of radon treatment related to pain reduction [87,88,89,90].

At present, the main application of radon for therapy is inhalation at former mines or bathing in radon-containing water (Appendix A). As the application procedures and indications for treatments expanded, the EURADON (European Association Radon Spas e.V.) was founded and started to define the indications for radon application, i.e., musculoskeletal and chronic pain diseases as well as pulmonary and gynaecological diseases (see Table 2).

**Table 2:** List of recommended indications for radon treatment [91].

Musculoskeletal disorders and chronic pain diseases	Ankylosing spondylitis and other spondylarthropathies (AS)
	Chronic polyarthritis (rheumatoid arthritis, RA)
	Chronic arthritis urica
	Psoriasis arthropathy
	Polymyalgia rheumatic
	Arthrosis and osteoarthritis (OA)
	Degenerative diseases of the spinal column
	Auxiliary treatment consecutive to intervertebral disc operations
	Osteoporosis
	Non-inflammatory soft tissue rheumatism (e.g., fibromyalgia)
	Chronic consequences of casualty or sporting injuries
	Auxiliary treatment consecutive to orthopedic operations
	Neuralgia, neuritis, polyneuropathy
	Multiple Sclerosis (MS)
Cutaneous disorders and diseases	Insufficiently healing wounds (e.g., ulcus cruris)
	Atopic dermatitis (neurodermatitis)
	Psoriasis
	Scleroderma
	Low grade circulatory problems of the skin
Pulmonary diseases	Asthma bronchiale
	Chronic-obstructive pulmonary diseases (COPD)
	Rhinitis allergica
	Chronic sinusitis
Gynaecological diseases	Praeclimacteric and climacteric disorders
	Pelvipethia spastica

### 4.1. Clinical Trials

In Europe and the United States, radon therapy is under ongoing discussion [92] because many historical trials were not in accordance with today’s evidence-based medicine [93]. Especially before 1993, studies did not include control groups or were not randomized. Between 1993 and 2000, only three prospective studies including radon therapy for patients with rheumatic disease were reported [94,95,96], all of them in German, and one is published as a PhD thesis. Lind-Albrecht investigated the effect of radon treatment in gallery (speleotherapy) versus sauna therapy in ankylosing spondylitis (AS) patients (*n* = 100, nonblinded) and found significant differences in pain reduction between the groups three months after the end of therapy [94]. Pratzel and co-workers [95] investigated pain parameters in a group of patients (*n* = 46) suffering from disorders of the cervical spine up to three months after the end of treatment. In this blinded and randomized study, patients were treated by bathing in radon-containing water (or tap water) (balneotherapy) and a long-lasting pain reduction (up to 3 months) was found only in the radon group. Later on, using the same conditions, the authors reported similar effects for patients with degenerative spinal disorders and osteoarthritis (OA) (*n* = 52) [96].

Due to the scarce database, clinical trials are seriously needed that are conducted according to the rules of global evidence-based medicine [97]. Unfortunately, the number of prospective, randomized, and blinded clinical trials performed, starting from 2000 with a reasonable group size is limited (Table 3). One major problem is the blinding of radon treatment as it is not possible to have a radon-free “sham gallery” for speleotherapy to efficiently separate a radon effect from a placebo effect. Accordingly, radon bathes are more eligible, because they can be applied in a blinded manner.

Therefore, three trials by Franke and colleagues, performed between 2000 and 2013, examined in a prospective and blinded manner the effect of radon/carbon dioxide (CO_2_) bathes on patients suffering from rheumatoid arthritis (RA) [98]. Sixty patients medicated with anti-rheumatic drugs were offered 15 bathes within four weeks with radon/CO2 water (radon activity of 1.3 kBq/L) or only CO_2_-containing water as a control. In addition, the patients had different manual therapies during the bath period and follow-up. Interestingly, both treatment groups had similar early effects, but the effect of pain relief lasted significantly longer in the radon group (up to six months), and confirmatory analyses showed a significant superiority in patients receiving radon balneotherapy [98]. In a subsequent randomized trial published in 2007, 134 patients were enrolled to radon/CO_2_ or CO_2_ balneotherapy only, similar to the first trial [99]. These patients showed no significant difference in pain intensity by visual analogue scale (VAS) between the treatment regimes, but differences increased with increasing follow-up time (up to nine months). In line with that, the confirmatory analysis showed a clear and significant effect of radon balneotherapy: the pain relief lasted longer in the radon group. In addition, drug intake was diminished in this group, resulting in a higher quality of life. However, these trials lacked an effective blinding of the water and were biased, since patients were at a regimen at the health resort during radon application. Further, these patients were allowed to have various manual therapies, whereas the control group had to stay at home [99].

The third trial of Franke et al. [76] addressed the above-mentioned bias problems partially. It was the first multicentric trial with 652 patients treated at different spas in Germany and Austria. This study called IMuRa was prospective, randomized and blinded. Patients suffering from OA, RA, AS and back pain (BP), received 12 bathes either with radon-containing water or the site-specific placebo (i.e., tap water, thermal water, or CO_2_ thermal water). The superiority of radon in inducing pain-relieving effects was confirmed and the intake of non-steroidal anti-rheumatic drugs (NSARDs) was significantly reduced in radon-treated patients for up to six months. The patients suffering from BP and inflammatory rheumatism (combination of RA and AS in this study) did not benefit from the radon baths as much as patients with OA did in terms of functional capacity.

Based on these findings, the GREWIS alpha consortium (funded by the German ministry of Research,02NUK050) started to analyze the contribution of the immune system in radon therapy responsiveness. By this, the RAD-ON01 trial was set up to analyze immunological alterations induced by radon balneotherapy in an explorative manner. One hundred patients enrolled in this study received either nine full radon bathes (1.2 kBq/L) or radon/CO_2_ bathes (0.6 kBq/L), respectively, in a covered bathtub to minimize radon inhalation. The bathing was double-blinded and whole blood of the patients was analyzed before, during, and at several time points after radon spa by detailed immunophenotyping, getting first hints for immunological markers of pain, bone destruction and inflammation [100,101,102], as described in more detail below. Similar to the trials described before, a significant pain reduction was quantified by VAS and pain dolorimetry for up to 18 weeks, performed at eight different tender points [103,104].

Several prospective, non-blinded trials conducted with patients at radon galleries were published. Van Tubergen and colleagues recruited 120 AS patients for three weeks of daily treatment in the radon gallery (speleotherapy with hyperthermia, HT) or “normal” steam sauna [105]. These patients also performed physical exercises. Since the patients of the two groups were not supposed to meet, the treatments were conducted at two different spa resorts in Europe. The patients who visited the radon gallery reported a significant and long-lasting ease of their pain. But these positive results could only be detected in a secondary analysis, since the power of the primary study goal (e.g., Bath Ankylosing Spondylitis Functional Index (BASFI), well-being, VAS-score) was too low to show statistical significance. Only a ‘pooled index of change’ analysis resulted in a significant beneficial effect for AS patients, which lasted up to 40 weeks after the spa-exercise program [105].

Another longitudinal observation of 33 AS patients revealed a significant reduction in the main AS scores, but the study was defined as a pilot trial lacking a control group [106]. Notable effects are described by a significant reduction of pain and enhanced functional behaviour in AS patients [107]. Interestingly, Dischereit et al. reported similar results in a trial with 48 patients (half/half of RA/AS, no blinding or control group) [108]. Here, patients with RA had more benefit from radon application, since the pain-relieving capacity lasted up to three months, while the effects in AS patients were diminished after three weeks [108]. A meta-analysis of several trials pointed out that the observed effects seem to be significantly triggered by bone restoration following radon exposure [109].

In summary, several trials starting from the year 2000 suggested that radon therapy has beneficial effects on patients with painful, degenerative, and inflammatory diseases describing a significant reduction of pain and enhanced mobility as well as increased quality of life. Other indications, singularly analyzed and based on small patient collectives or historic cohorts do not seem to be adequately proven, like dermal inflammatory diseases [110], fibromyalgia [111], and respiratory diseases [112].

**Table 3:** Clinical trials with radon application from year 2000 on.

First Author Year of Publication	Trial Design	Patient Number Indication	Dose	Type of Exposure Frequency Duration	Endpoints and Timepoints	Most Important Findings	Ref.
Franke et al., 2000	Prospective; blinded; randomized	60 patients RA	**Radon group:** 1.3 kBq/L, 1.6 g/L CO_2_ **Placebo group:** 1.6 g/L CO_2_	Bath 20 min 15 times 4 weeks T = 35 °C **Additional:** Physiotherapy Occupational therapy Galvanic bathes (3/week) Classic massage	**Endpoints:** Pain intensity (VAS) Keitel functional Test (KFI) Arthritis Impact Measurement (AIMS) **Timepoints:** Before and directly after therapy, as well as, 3 and 6 months after therapy.	Pain intensity decreased in both groups, radon treatment results in a significant and longer lasting benefit from pain relief; KFI more in radon group; AIMS score was significantly increased in radon treated patients up to 6 months; KFI score shows a not significant benefit in radon treated patients	[98]
Van Tubergen et al., 2001	Prospective; different treatment groups at different places.	120 patients AS (40 spa with radon, 40 spa w/o radon 40 physical therapy at home)	**Radon group:** 0.536 WLM **Placebo group:** Thermal water + sauna Hydrotherapy Bathing Sports	Gallery/ inhalation Each 1 h 16 times 3 weeks T = 38–41 °C **Additional:** Physical exercise Postural correction therapy	**Endpoints:** BASFI Well-being VAS Pain intensity VAS Morning stiffness **Timepoints:** Before therapy After therapy week 4, 16, 28, and 40	Primary goals borderline significant; pooled index of change shows highly significant differences as well as long-lasting effects of radon compared to conventional treatment	[105]
Yamaoka et al., 2004	Prospective	15 people (putative healthy individuals)	**Radon group:** 2080 Bq/m^3^ **Sauna Group:** 54 Bq/m^3^ **Control Group:** 54 Bq/m3	Inhalation 40 min 5 times T_Radon_ = 36 °C T_Sauna_ = 48 °C T_Control_ = 36 °C	**Endpoints:** SOD AOC lipid metabolism CD4/CD8 immune cells vasoactive substances diabetes-associated markers **Timepoints:** Blood draw before and at 2 h after each treatment. In addition, 5 and 10 days after treatment.	Significant increase in SOD as well as decrease of lipid metabolism and cholesterol at 10 days for radon and sauna group; radon enhances T cell activity significantly, while sauna has similar effects, only significant at 10 days after treatment; radon enhances the CD4 T cell amount significantly after treatment, while CD8 T cells were decreased, respectively; radon group shows significantly more endorphin and a reduced vasopression	[112]
Yamaoka et al., 2004	Prospective	20 patients OA	**Radon group:** 2080 Bq/m^3^ **non-controlled**	Inhalation 40 min each Every 2 days T = 42 °C	**Endpoints:** SOD, catalase, lipid peroxide, total cholesterol, GSH, β-endorphin, ACTH, uric acid, ANP, and vasopressin levels in blood **Timepoints:** Before therapy, 2 h, 2, 4 and 6 weeks after therapy	SOD activity is significantly and long-lasting increased; Catalase activity is significantly increased after 4 and 6 weeks; T cells of CD4 type are increased, while CD8 T cells are decreased from 2 to 4 weeks after therapy; β-endorphin and anti-ANP levels were significantly and long-lasting increased after therapy; Vasopressin was significantly and long-lasting decreased; Cholesterol and lipid peroxide levels were significantly and long-lasting decreased	[113]
Shehata et al., 2006	Retrospective	83 patients AS (radon treatment) 10 patients AS (conv. Treatment) 10 patients LBP	**Radon group:** ~4.5 nCi/l **Placebo groups:** Convent. Therapy	Gallery/ inhalation 1 h each T = 38–41 °C 9–12 times 3–4 weeks **Additional:** Physiotherapy Hydrotherapy Massage Exercises	**Endpoint:** TGF-β (total and active form) **Timepoint:** Before, during and after the treatment (~0, 2 and 4 weeks)-	Total TGF-β level increased significantly in radon exposed patients compared to conventional treated patients or LBP controls; active TGF-β increased strongly and significantly in radon exposed patients compared to conventional treated patients or LBP controls; therapy responders show an inverse correlation with CRP concentration	[107]
Franke et al., 2007	Prospective; blinded; randomized	134 patients RA (67 patients per group)	**Radon group:** 1.1 kBq/L, 1.3 g/L CO_2_ **Placebo group:** 1.6 g/L CO_2_	Baths 20 min 15 times 3 weeks T = 35 °C **Additional:** Physiotherapy Occupational therapy Galvanic bathes (3/week) Swedish massage	**Endpoints:** pain intensity, pain frequency, morning stiffness, functional capacity (all VAS), Drug intake **Timepoints:** Before and after last treatment, 3, 6, 9, and 12 months after treatment	Drug intake was significantly reduced from month 9 on; both groups had treatment effects, most not significant; repeated measurement ANCOVA revealed significant and long-lasting enhanced quality of life due to fewer limitations induced by pain	[99]
Moder et al., 2010	Prospective	33 AS patients	**Radon group:** ~4.5 nCi/L **non-controlled**	Gallery/ inhalation 90 min each 10 times 3 weeks 37–40.5 °C	**Endpoints:** Disease activity, BASDAI. BASFI, BASMI serum concentration of RANKL, OPG, TNFα, TGF-β, IL-17, IL-6 **Timepoints:** Before and after therapy (3 weeks)	Disease-associated scores BASDAI. BASFI, BASMI decreased significantly after therapy; serum conc. of TGF-β1, IL-6, TNF-α, RANKL, OPG, as well as OPG/RANKL ratio was significantly increased; active form of TGF-β, IL-6, TNFα.	[106]
Franke et al., 2013 (IMuRa Trial)	Prospective; blinded; randomized; multicentric	652 Patients BP 437 pts. OA 230 pts. RA 98 pts. AS 39 pts. Multi 146 pts.	**Radon group** (332 patients) Radon bathes according to specific center (with or without CO_2_) or Radon Speleotherapy **Control group:** (320 patients) Placebo bathes according to specific center (either tap water or non-radon-containing fountain, with or without CO_2_)	Bath 20 min 12 times 3–4 weeks T = 36–38 °C	**Endpoints:** Pain intensity (VAS) Pain Questionnaire Functional capacity (FFbH) Western Ontario questionnaire (WOMAC) Health assessment questionnaire (HAQ) BASFI Drug intake **Timepoints:** Before and after last treatment, 3, 6, and 9 month after treatment	Radon treatment leading to significant and long-lasting relieve of self-assessed pain (VAS); OA and BP patients have the strongest and most lasting benefit from radon treatment, while OA patients seem to additionally have an enhanced quality of living up to 6 months after treatment	[76]
Dischereit et al., 2014 (Article in german)	Prospective	24 patients RA 24 patients OA	**Radon group** 44 kBq/m^3^ **non-controlled**	Gallery/ inhalation 60 min each 12 times 3 weeks T = 37.5–41 °C	**Endpoints:** Pain intensity and duration Disease activity and functional score (BASDAI; BAS-G) Serum levels of RANKL, OPG, and TNF-α **Timepoints:** Directly before and after therapy, as well as 3 months after therapy	Pain was relieved after therapy and after 3 months in AS patients and after 3 months in OA patients; BASDAI was reduced significantly, and long-lasting in AS patients; TNF-α level was decreased in both groups, significantly in OA; RANKL level was significantly decreased in both groups, OPG increased only in AS; RANKL/OPG ratio decreased only AS significantly	[108]
Winklmayr et al., 2015	Prospective; blinded; randomized	64 healthy individuals Married partners	**Radon group** 412–900 Bq/L, Placebo: thermal water	Bath 20 min 5 times + 3 times brush up T = 36–39 °C **Additional:** Mountain hiking 3–4 h daily	**Endpoints:** Serum conc. OPG, RANKL, OPG/RANKL ratio **Timepoints:** Day 0, 6, 60, and 63 and 6 months after last treatment	Treatment benefits were seen in both groups in OPG, RANKL, and OPG/RANKL ratio; detected borderline significant trend towards bigger effect in Radon treated group	[114]
Lange et al., 2016 and 2012	Prospective	25 patients RA 24 patients OA	**Radon group** 4.5 nCi/l **non-controlled**	Gallery/ inhalation 60 min each 12 times 3 weeks T = 37.5–41 °C	**Endpoints:** serum conc. RANKL, OPG, TNF-α, and ACPA **Timepoints:** Directly before and after therapy	The serum conc. of TNFα and RANKL levels decreased in both groups; only in RA patients, OPG level increased, leading to a decreased RANKL/OPG ratio; ACPA titers decreased only in RA patients	[115,116]
Lange et al., 2017					**Endpoints:** Pain VAS FFbH questionnaire ESR Serum CRP, RANKL, OPG, TNF-α, IL-10, and ACPA **Timepoints:** Directly before and after therapy, as well as 3 months after therapy	RA patients have significant immediate and lasting effect in pain relief, while health status (FFbH) is increasing; OA patients have significantly lasting pain relief effect; serum concentration of IL-10 is significantly increased directly after treatment in RA patients	[117]
Rühle et al., 2017 (RAD-ON01)	Prospective Blinded Randomized	100 patients with musculoskeletal disorders 50 patients per group **Ambulant patients**	**Radon group** 1200 Bq/L, Radon water only group); **Radon/CO_2_ group** 600 Bq/L and 1 g/l CO_2_; Radon-CO_2_-group Covered bath-tube	Bath 20 min each 9 times 3 weeks T = 35 °C	**Endpoints:** Immune modulation via DIoB [100] method Pain relief (VAS and questionnaire) Pain sensitivity (dolorimetry, pressure point measurement) **Timepoints:** Directly before as well as 6, 12, and 30 weeks after therapy	Long-lasting and significant pain reduction until end of observation period in whole trial population; significant and long-lasting increase in T cells and monocytes; significant temporarily increase of dendritic cells and regulatory T cells; significant and long-lasting reduction of the expression of the activation marker CD69 on T, B, and NK cells	[104]
Cucu et al., 2017 (RAD-ON01)					**Endpoints:** Amount of regulatory T cells Serum markers of bone and lipid metabolism	significant and long-lasting decrease of collagen fragments (CTX-I) and reduced levels of visfatin. Both factors are correlating significantly with pain intensity (VAS); regulatory T cells increase significantly and long-lasting after treatment	[102]
Rühle et al., 2018 (RAD-ON01)					**Endpoints:** Pain relief (VAS and questionnaire) Pain sensitivity (dolorimetry, pressure point measurement) Blood pressure Antioxidative capacity (AOC) Superoxiddismutase (SOD)	Long-lasting and significant pain reduction until the end of the observation period in whole trial population, Radon CO_2_ bathes show a trend to be less effective (n.s.); lowered blood pressure in both groups, nightly measured systolic and diastolic blood pressure significantly decreased in Radon/CO_2_ treated patients; SD-VLF decreased significantly after radon therapy; SOD2 reduced significantly 6 weeks after treatment and increased significantly long-lasting	[103]
Kullmann et al., 2018 (RAD-ON01)					**Endpoints:** Detection of inflammatory and anti-inflammatory cytokines in serum of patients.	No significant effects found for TNFα, IL-1β, IFNγ, IL-18, IL-1Ra, IL-10 concentration in serum of the patients; TGF-β concentration was significantly increased after treatment and significantly correlates with pain sensitivity; IL-18 level corresponds with lowered pain perception	[101]

Abbreviations: ACPA: anti-citrullinated peptide antibodies; ACTH: adrenocorticotropic hormone; ACTH: Adrenocorticotropine; AIMS: arthritis impact measurement score; ANP: atrial natriuretic polypeptide; AOC: Antioxidative Capacity; AS: ankylosing spondylitis; BAG-G: Bath Ankylosing Spondylitis Patient Global Score; BALF: bronchioalvelolar lavage fluid; BASDAI: Bath Ankylosing Spondylitis Disease Activity Index; BASFI: Bath Ankylosing Spondylitis Functional Index; BASMI: Bath Ankylosing Spondylitis Metrology Index; BP: Back Pain; CD: cluster of differentiation; CO_2_: carbon dioxide; CRP: c-reactive protein; CTX: Cross Laps; FFbH: Funktions Fragebogen Hannover (Functional Capacity); GSH: Glutathione; HAQ: Health assessment questionnaire; IFN: interferon; IL: interleukin; KFI: Keitel functional index; LBP: lower back pain; OA: Osteoarthritis; OPG: osteoprotegerin; RA: rheumatoid arthritis; RANKL: receptor activator of NFkB Ligand; SOD: superoxide dismutase; TGF: transforming growth factor; TNF: tumor necrosis factor; VAS: Visual Analog Scale; WOMAC: Western Ontario questionnaire. Bold letters highlight the trial groups, additional treatments, trial endpoints, and timepoints of investigation.

### 4.2. Biomedical Investigations in Patients

In addition to the evaluation of pain or functionality of joints, the biomedical investigations reviewed in the following paragraph revealed treatment-induced changes of the immune status and release of specific factors. These are cytokines, hormones and growth factors, which are known to influence pain perception, inflammation, bone metabolism and the cardiovascular system.

One putative key player associated with pain reduction is the anti-inflammatory cytokine transforming growth factor beta 1 (TGF)-β1. Indications come from patient studies, all not blinded and without control groups. In AS patients undergoing combined radon speleotherapy and exercise treatment, an increase of serum levels of both, the precursor and activated TGF-β1 was detected directly after therapy while this was not the case for lower back pain patients [*n* = 83, prospective study] [107]. For a subgroup of “responders” [*n* = 48], a correlation of morning stiffness and decreased C-reactive protein (CRP) level was observed directly after therapy, suggesting that the pain reducing effect of TGF-β1 is based on a reduction of inflammation [108]. A comparable increment in the serum levels of active TGF-β1 was found directly after therapy for different treatment modalities and diseases, i.e., in the serum of AS patients [*n* = 33] after radon speleotherapy [106] and six weeks after radon balneotherapy, in a larger cohort of patients [*n* = 100], suffering from non-rheumatic, musculoskeletal diseases (MSD) [101].

Studies on β-endorphin, another important signaling protein, are also pointing to a reduced pain perception after radon treatment. Levels of β-endorphin were found to be increased directly after radon speleotherapy in OA patients [*n* = 15, control group: sauna] [113] and slightly (not significant) in patients with chronic respiratory diseases [*n* = 81] [118].

In addition, inflammation, which is likely to be a cause of pain, was investigated. Regardless of a chronic or acute inflammatory status of the patients before treatment, low serum levels of the pro-inflammatory cytokines tumor necrosis factor (TNF)-α, interleukin (IL)-1β, interferon (IFN)-γ and IL-18 were detected. For example, despite low basal TNF-α levels, they further decreased significantly in OA and RA patients after combined radon and HT treatment [OA: *n* = 48, balneotherapy [108]; RA: *n* = 49, speleotherapy [116]; sample collection directly after therapy]. A clear anti-inflammatory effect in RA patients was confirmed in one of these studies based on the levels of ACPA (Anti–citrullinated protein antibodies) along with inflammatory cytokines and pain reduction [116]. In contrast, for AS patients, the TNF-α decrease was less pronounced as reported in the study of Dischereit and co-workers [108].

Decreased serum levels of IL-18 were observed in MSD patients [mostly OA, *n* = 100] directly after radon balneotherapy and correlated with reduced pain perception [101]. However, only a trend was observed, and the treatment was radon exposure alone, suggesting that the anti-inflammatory effect is relatively weak and becomes more pronounced in combination with HT. This idea is endorsed by the results of a study performed in AS patients for radon and HT- speleotherapy [*n* = 33], where disease scores were improved and TGF-β1 was increased [106]. A weak point of this study is that the serum levels were measured only directly after exposure.

The studies as mentioned above, however, all have to be interpreted with care as they were non-blinded and mainly lack control groups. In line with that, a potential causal relationship of β-endorphin and TGF-β1 levels remains to be elucidated. However, increasing evidence is provided for treatment-induced changes in the immune status of the patients. In an earlier study with a low number of patients enrolled [*n* = 15] a combined treatment with radon and HT was compared to HT alone. Proliferation of CD4+ T-helper cells was increased after ex vivo stimulation, whereas the response to stimulation with concanavalin A of CD8+ cytotoxic T-cells was decreased. Both effects were lasting until the end of therapy (10 days) only in the radon-HT-group, but not in the group receiving HT only [112]. The interpretation of these treatment-induced changes is difficult, as there are not enough data on the interaction of immune cells. More recently, a broader view of the immune status of MSD patients was provided in the frame of a larger study where a detailed immune phenotyping was performed after radon balneotherapy [*n* = 100, RAD-ON-01 study]. While the large immune cell classes such as B-cells or T-cells remained almost unaffected, the results suggest transient anti-inflammatory and immune inhibiting effects. For example, mostly immune-suppressive regulatory T cells (Treg) were increased up to 12 weeks in the complete cohort [104]. In addition, Treg levels that were investigated in a smaller subgroup of this large cohort remained increased over the whole observation period of 30 weeks, whereas the amount of immune-stimulating T helper cells (Th17) was not changed [102]. In addition, common activation markers like CD69 and HLA-DR were altered and stayed upregulated (HLA-DR) or downregulated (CD69) during the observation period.

Since radon-treated patients reported improvements in mobility, diagnostic markers for bone formation (OPG, osteoprotegerin) and bone resorption (RANKL, receptor activator of nuclear factor kappa b ligand) were studied. A positive influence of a combined radon and HT-balneotherapy on bone metabolism was investigated in a randomized and blinded trial. This trial enrolled postmenopausal women who were healthy but at risk for developing osteoporosis [*n* = 64, randomized, blinded, controlled]. A control group received regular water bathes; both groups underwent regular physical exercise. A slight increase of the OPG/RANKL ratio was observed in both treatment groups, lasting up to two months only after radon treatment, indicating enhanced bone formation and/or reduced bone resorption. However, these changes, along with the observed increase of other markers for bone formation (osteocalcin and osteopontin), cannot be attributed to radon treatment alone because of the combination with enhanced physical exercise during treatment [114]. In AS and OA patients, hints for changes in bone metabolism were obtained in studies without physical exercise, after combined radon and HT speleotherapy treatment. RANKL serum levels were significantly decreased in these patients directly after therapy [*n* = 48] [108]. In a second study, the same authors report similar results for RA patients in combination with decreased disease activity and functional restriction, and increased spine mobility score directly after therapy [117]. Taken together, for AS and RA patients, the indications for reduced bone resorption and, in some cases, enhanced bone formation are reported [119,120]. In line with the above-mentioned weaker effect reported for MSD (mostly OA) patients [*n* = 32], no significant alterations of RANKL and OPG after radon balneotherapy were found for up to 30 weeks after therapy. However, a reduced bone resorption can be assumed because collagen fragments (CTX-I) in serum samples were significantly lower during the 30-week period of biomedical follow-up [102].

In the following, some smaller studies are reviewed in order to highlight single observations concerning adipokines related to chronic inflammation, pain-related stress hormones, antioxidative capacity, and the cardiovascular and central nervous system. Those findings, substantiated by most studies, may contribute to clarify the mechanism of action of radon therapy after verification in larger patient cohorts.

Some hormones, i.e., leptin and visfatin, are typically released by the adipose tissue and play a role in the pathogenesis of chronic inflammatory bone diseases [121]. Changes of these adipokines after radon treatment were recently published [102]. Following radon balneotherapy alone, visfatin levels were found to be significantly reduced over the observation period of 30 weeks in MSD (mostly OA) patients [102]. One of the aforementioned studies [114], where radon balneotherapy or bathes in normal water were combined with physical exercise, revealed decreased leptin levels, concomitantly with increased osteocalcin levels.

Pain is a stressor activating the hypothalamic–pituitary–adrenal–thyroid–gonadal (HPATG) system, which includes hormones like cortisol, insulin, thyroid hormones, or adrenal corticotropin hormone (ACTH) [122]. Reduced activation of these signaling molecules could be an indirect indication of a modified pain perception. A couple of studies were conducted, most of them for a combined treatment with radon and HT. Accordingly, the specific effect of radon treatment cannot be discriminated from these investigations yet.

Two studies with radon speleotherapy revealed that serum levels of insulin [*n* = 15] [112] and ACTH [*n* = 20] [113] were increased for OA patients, directly or two weeks after therapy, respectively. A decreased activation was found for thyroid hormones directly following radon speleotherapy alone, mostly in male patients with chronic respiratory diseases [*n* = 81] [112]. The treatment-induced changes in the regulation of these hormones may imply a role in the response to radon therapy, although analyses were restricted to short periods after the end of treatment only.

Also, after combined radon and HT balneotherapy, but in combination with physical exercise and in healthy individuals, adrenocorticotropic hormone (ACTH) was decreased over the course of follow-up of 6 months, [*n* = 53, blinded, randomized, placebo controlled]. In addition, a long-lasting decrease of parathyroid hormone (PTH) serum levels in both treatment groups (HT balneotherapy with or without radon) was reported. PTH indirectly stimulates osteoclast activity in bones [114], indicating an additional reason for the putative decrease of bone resorption after treatment.

Hints for a beneficial impact of radon therapy on the cardiovascular system were also reported. In the RAD-ON-01 balneotherapy study, all patients had lowered blood pressure, a long-term relaxation effect and decreased heart rate variability. These effects indicate a modulation of the sympathetic nervous system and a relaxation of smooth muscles in the cardiovascular system [103]. In a study of OA patients [*n* = 20], atrial natriuretic peptide (ANP), a vasodilator, was increased after speleotherapy [113], whereas vasopressin, a vasoconstrictor, was decreased [123], which could explain the effects.

Indications for an enhanced antioxidative capacity were obtained in two studies. One study showed, for combined radon and HT speleotherapy, a decreased lipid peroxide and cholesterol level, while superoxide dismutase (SOD) was increased in both treatment groups directly after treatment [*n* = 15] [112], indicating an enhanced antioxidative capacity. In MSD patients [*n* = 100, RAD-ON-01], the SOD levels were decreased at early time points (6 weeks), but increased later after radon balneotherapy [103], emphasizing the importance of longitudinal assessments of treatment-induced changes.

### 4.3. Animal Studies

Although radon therapy is in therapeutic use for decades, preclinical studies on underlying mechanisms are scarce and restricted to the last 20 years. The few studies available will be summarized in this paragraph. The review, however, will exclude lung cancer studies, performed in rats after radon exposure [124] because these investigations highlight the effects of chronic exposure.

Although well conducted, the design of most studies investigating non-cancer effects of radon treatment challenges their relevance for the impact of patient treatment. No animal studies are available investigating the effects of the typical exposure situations, such as radon bathing or using animal models for the main indications of radon therapy, i.e., rheumatoid arthritis and Morbus Bechterew. Furthermore, the experimental design of these studies hardly overlaps with treatment conditions. Nevertheless, some basic information about the activation of antioxidative mechanisms can be inferred from these studies. In some of the disease models, an enhanced SOD activity and higher t-GSH levels in the blood and different organs were found [125,126,127,128], which is in line with the measurements in OA patients mentioned above [103,112]. Interestingly, an enhanced antioxidative activity was also observed in healthy mice [129,130], thus pointing to a more general mechanistic feature of radon exposure.

Using a polyarthritic mouse model to investigate the clinical effects of radon exposure, ongoing experiments investigate the underlying mechanisms and their potential correlation to radon exposure. In the same mouse model, beneficial effects of low dose radiotherapy with photons have already been reported [131]. Furthermore, experiments to test the effect of radon on chronic inflammatory skin diseases, i.e., psoriasis in a mouse model, are performed. Notably, for treatment of psoriasis, no animal or valid patient studies are published up to now, although the disease covers an indication for radon spas and speleotherapies (see Table 4). However, in one animal study, the impact of radon exposure on atopic dermatitis, which also covers an indication for radon treatment, is assessed [132]. The authors reported significantly lowered severity score of the skin lesions, together with a lower immunoglobulin E (IgE) level after radon treatment. Importantly, these beneficial effects were only found after pre-treatment with radon prior to skin sensitization with picrylchloride, indicating a protective rather than a curing effect of radon treatment. From a mechanistic point of view, this is endorsed by other animal studies (Table 4), where radon treatment was also started before disease induction.

Abbreviations: WT: Wild type, PO: Potassium oxonate, UI: Ulcer index, IHI: Index of histological injury, SOD: Superoxide dismutase, XOD: Xanthine oxidase, CAT: Catalase, GPx: Glutathione peroxidase, GR: Glutathione reductase, GOT: Glutamic oxaloacetic transaminase, GPT: Glutamic pyruvic transaminase, ALP: Alkaline phosphatase, CRE: Creatinine, T-CHO: Total cholesterol, LP: Lipid peroxidase TG: Triglyceride, AA: L(+)-ascorbic acid, TNF-α: Tumor necrosis factor alpha, t-GSH: Total glutathione content, NO: Nitric monoxide, CCI: Chronic constriction injury, NIK: NF-κB–inducing kinase, IKK-β: Inhibitor of κB kinase-β, ATM: Ataxia-telangiectasia mutated kinase, MPO: Myeloperoxidase, DAI: Disease activity index, WLM: Working level months, hprt hypoxanthine phosphoribosyl transferase, MNR: Micronuclei rate.

## 5. Discussion: What Do We Know So Far about the Dose Distribution and Mechanism of Action Originating from Radon Exposure and Where Are Limitations?

When considering the physical and biological interaction of radon with the human body, large uncertainties are emerging. This is mainly due to the fact that there are only fragmentarily data available for radon distribution in the human body and on underlying biological mechanisms. For radiation protection purposes related to occupational and indoor radon exposure, knowledge of the physical characteristics and the morphometry and physiology of the respiratory tract has been combined to model dose deposition in the lung and in inner organs. Models predict that the lung equivalent dose makes up for over 95% of the effective dose, whereby over 95% of that dose is caused by progeny and less than 5% by the radon gas itself. Besides the lung, organs with a high fat content receive the highest dose due to the high radon solubility in those tissues [1,43,63,149]. Still, models cannot consider all variations in external environmental conditions and individual physiological factors, but can discriminate between typical exposure scenarios, leading to a more exact dose determination in individual cases. However, the experimental database for model calculations of the distribution of incorporated radon and thus energy deposition in the body are based on data obtained from just a handful of studies performed decades ago, making further investigations for a proper dose determination necessary [51,56,58]. In biokinetic models, an estimation of cancer risk is based on dose conversion factors, as specified in ICRP 137 [1]. Only recently, investigations on radon relevant for the estimation of cancer risk have restarted with state-of-the-art technologies [55,150]. Major target organs of radon exposure, i.e., lung and adipose tissue, have been confirmed [1,31,62,63]. However, further extension of the experimental database is still desirable to fully elucidate target tissues and organs.

In epidemiological studies, cancer risk related to chronic exposure (occupational, indoor) has been evaluated, providing data sets allowing for estimations of the lung cancer risk based on activity concentrations. These estimations are valid, but at low activity concentrations, the uncertainties are significantly high. Despite large uncertainties at low activity concentrations, a cancer risk from radon exposure cannot be denied. Albeit model approaches assuming a non-linear dose-response relationship for low radiation doses, such as ‘hormesis’ are discussed, but large and sufficiently powered epidemiological studies on lung cancer risk following chronic radon exposure show a linear dose-response relationship without threshold dose [68,69,71,73]. For non-chronic exposure scenarios that are relevant for radon therapy of chronic inflammatory diseases, epidemiological data to estimate the cancer risk are completely lacking. As pointed out the additional uncertainties especially to long-term drug intake also complicates the analysis of a reliable value for the excess risk of radon therapy by radon itself. So, there is an urgent need of prospective and quality-controlled trials to analyze these hypotheses. In spite of this, a high number of patients expose themselves to radon, because they experience a benefit from the treatment. The therapeutic efficacy of radon therapy to ameliorate the symptoms of patients with chronic, degenerative and painful diseases is significant and the major goals are achieved, i.e., higher mobility and pain alleviation [76,98,99]. Thus, it is reasonable to assume, but not proven that the ratio of risk and benefit related to a radon therapy is different for the patients compared to healthy individuals.

Besides the above-mentioned uncertainties for the distribution and thus dose application of radon in the human body and the associated risk, radon is used for decades for the therapy of inflammatory diseases. In view of these uncertainties, the discussion about radon application in patients with chronic diseases will continue. In line with that, there is an urgent need for more quality controlled clinical trials for radon treatment to obtain a higher level of evidence as well to obtain reliable data on the risk of radon itself in therapeutic application. For example, the level of evidence for the efficiency of radon bathes was set to a moderate level in the Cochrane report by Verhagen et al. [93]. For radon balneotherapy an effective blinding is possible reducing the patients’ bias. Newly designed trials should always include safety analyses to get a balanced view on this type of treatment (risk-benefit-analyses). Currently, two major trials are running addressing many of the above- mentioned problems:

(I) The RAD-ON02 trial (EudraCT: 2016-002085-31; DRKS00016019) according to the German drug law was started in 2018 and covers molecular and osteoimmunological analyses correlated to pain relief as well as safety issues of the patients treated in radon bathes. The final analysis of this placebo-controlled, blinded, and randomized trial is anticipated for late 2021 [151].
(II) The radon register trial of Austria was started in 2017 to cover the procedures and effects of many patients as a European basis for upcoming multicenter trials [152].

**Figure 2:**
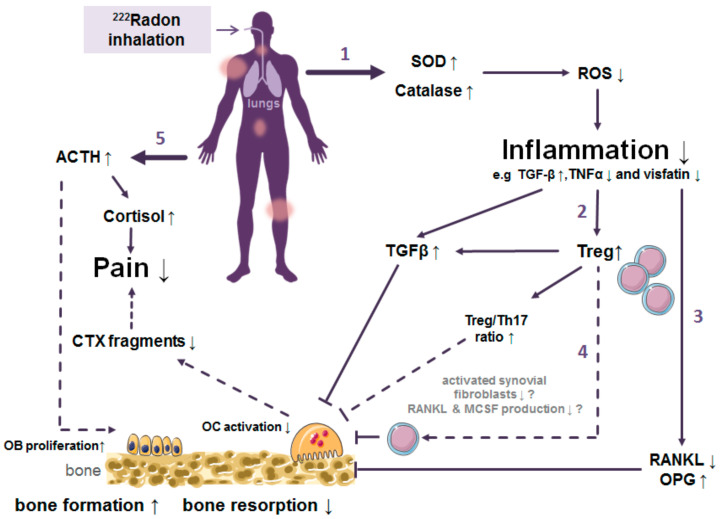
Proposed mechanism of action when radon is used to treat patients with a treatment for chronic musculoskeletal diseases (mostly ankylosing spondylitis, osteoarthritis or rheumatoid arthritis). Findings from in vitro or patient studies have been combined in this graph, where a solid line represents experimental findings (─) and a dashed line indicates a hypothetical relationship (- - - -). Please see the text for a more detailed discussion and the explanation of the proposed mechanisms (indicated by the numbered arrows). Abbreviations: ACTH Adrenocorticotropic hormone; CTX collagen fragments type I; OC osteoclasts; IL interleukin; RANKL receptor activator of nuclear factor-κB ligand; OPG osteoprotegerin; ROS reactive oxygen species; SOD superoxide dismutase; TGF transforming growth factor; ↓ means decrease, while ↑ means increase. Illustrations based on pictures from Smart Servier Medical Art under the Creative Commons Attribution 3.0, France.

However, in contrast to the efficacy of a radon treatment, a scientific basis for the causative relationship between beneficial effects of radon treatment and the concomitant radiation exposure is still needed. In this review, we aimed at summarizing the current knowledge on putative underlying mechanisms and causal relationships, thereby highlighting hypothesis and preliminary versus established results. According to the results on biomedical investigations reported in this review, we suggest a multifactorial effect of radon exposure on the course of the disease in radon exposed patients. This is illustrated in Figure 2:

(1) Trigger of the antioxidative defence by increased superoxide dismutase (SOD) and catalase activities.
(2) Inhibition of the local and systemic inflammatory processes by increased release of TGF-β1 along with reduced TNF- α levels.
(3) Decreased activation of immune cells and shift of the ratio of immune cells towards a more anti-inflammatory state.
(4) Alterations in bone metabolism resulting in diminished bone erosion.
(5) Enhanced bone formation and pain release are mediated by hormones.

The primary route of radon intake is inhalation. Inhaled radon daughter nuclei attach to the epithelial surface and radon is distributed via diffusion and active transport to different organs. The main target organ therefore is the lung, but in bone marrow and fat tissue radon daughter nuclides also accumulate. In view of the clinical application and the biomedical results obtained in patients also the musculoskeletal system has to be considered. In MSD, bone and structures of the joints are affected by erosion or resorption, often accompanied by inflammatory processes [153]. It is plausible to assume that cellular reactive oxygen species (ROS) production is part of the pathogenesis of many of the diseases treated with radon, because it is followed by an inflammatory reaction, characterized i.e., by enhanced production of TNF-α and other cytokines [154,155]. For example, in MSD patients, TNF-α is involved in recruiting OC progenitors to sites of inflammation [156], as to the joints, resulting in an increased bone resorption. According to measurements in the serum of patients, the antioxidative defense is activated, i.e., SOD is increased after radon treatment (Figure 2; arrow 1) [103], which was also reported in animal studies [141]. ROS levels are difficult to measure directly, but the above-mentioned findings indicate a reduction after radon exposure. A concomitant reduction of the levels of pro-inflammatory cytokines such as TNF-α was reported in some patient studies (e.g., [108,116]). Remarkably one potential antagonist of TNF-α is the pleiotropic cytokine TGF-β1, which can also be activated by ROS [157]. In the types of diseases treated with radon this cytokine can either foster a pro-inflammatory immune reaction by inducing the differentiation of T cells into Th17 cells, together with IL-6 [158,159,160]; or, in contrast, lead to an up-regulation of anti-inflammatory Treg cells (Figure 2; arrow 2). As can be expected, TGF-β1 levels were found to be increased [101,107], and the ratio between Th17 and Treg cells was changed in the serum of patients upon radon balneotherapy, the latter mainly due to an increase of the amount of Treg cells [102,104], which possibly attenuates the inflammatory reaction and may also inhibit osteoclast activity [131].

In joints of patients suffering from autoimmune bone diseases, activated Th17 cells and also pro-inflammatory synovial fibroblasts produce the growth factors RANKL and MCSF, leading to an increased OC differentiation and bone resorption [156]. A decrease of RANKL release, most likely associated with a reduction of bone resorption by OC, has been shown after radon treatment of RA patients (Figure 2; arrow 3) [116] and is claimed also for AS patients [106]. Not only via the RANKL/MCSF axis, but also by an increased proportion of Treg cells, triggered by the aforementioned elevated TGF-β levels, bone resorption is impacted (Figure 2; arrow 4). This could probably be due to direct interaction of Treg cells with OC precursors via IL-4, IL-10, and TGF-β1 as well as cytotoxic T-lymphocyte-associated protein 4 (CTLA4)-signaling, shown in murine cells [156]. In the same line of evidence, in patient studies, the RANKL-antagonist OPG was found to be enhanced after radon balneotherapy. This finding supports the proposed reduction of bone erosion in MSD (mostly OA) patients [102,108,114,116]. Additionally, pathological bone erosion seems to be counteracted after radon treatment by new bone formation, which could be caused by a stimulating effect of radon therapy on ACTH production and an upregulation of cortisol. As a consequence, pain is reduced, and osteoblast proliferation is promoted (Figure 2; arrow 5) [112,114,161].

## 6. Conclusions

In summary, experimental research on the effects of radon exposure is needed on multiple levels. For risk assessment related to different exposure scenarios including therapeutic application, the estimations of organ doses and mechanisms of intake and elimination of radon and its progeny have to be underpinned with more solid experimental measurements. The clinical applications have to be further analyzed in high quality and placebo-controlled trials, accompanied by biomedical investigations, to increase the level of evidence of the therapy as well as for assessment of potential side effects. This will help not only the patients directly in enhancing their mobility, but also might have a positive socioeconomic effect for an aging population.

## Figures and Tables

**Table 1:** Decay scheme of ^222^Rn and ^220^Rn [22].

^222^Rn			^220^Rn		
Nuclide	Half-Life	Decay-Mode	Nuclide	Half-Life	Decay-Mode
^222^Rn	3.825 d	α	^220^Rn	55 s	α, γ
^218^Po	3.05 min	α	^216^Po	0.15 s	α
^214^Pb	26.8 min	β, γ	^212^Pb	10.64 h	β, γ
^214^Bi	19.9 min	β, γ	^212^Bi	60.6 min	α, β, γ
^214^Po	164 µs	α	^212^Po	304 ns	α
^210^Pb	22.3 a	β, γ	^208^Tl	3.05 min	β, γ
^210^Bi	5.0 d	β, γ	^208^Pb	stable	
^210^Po	138.4 d	α			
^206^Pb	stable				

**Table 4:** Animal studies with radon.

First Author Year of Publication	Species	Group Size	Type of Treatment and Dose	Time of Analysis after Exposure	Disease Model	Endpoints	Most Important Findings	Ref.
Takahashi et al., 2006	Mice (SPF NC/Nga, female, 5 weeks) Mice (C57BL/6, male, 6 weeks)	*n*= 4–9	Drinking water; 203 Bq/L; approximate amount of radon ingested by each mouse 140–176, 68–85 and 0.86–1.08 Bq/kg week	Up to 4 weeks	Atopic dermatitis model: sensitization with 5% purified picrylchloride Lung metastasis model: injection of B16 melanoma cells (both 2 weeks after start of radon treatment)	Atopic dermatitis: Skin severity score, Plasma IgE Lung metastasis: number of metastasis	Lower skin severity score and lower plasma IgE, only after radon pretreatment, Lower number of lung metastasis only after radon pretreatment and small number of inoculated tumor cells	[132]
Kataoka et al., 2011	Mice (BALB/c, male, 7–8 weeks, 25 g)	*n* = 5 (Exp.3) *n* = 4–7 (Exp.4) *n* = 5–6 (Exp.5)	Exp.3: inhalation for 24 h, 4000 Bq/m^3^ Exp:4 600 and 3500 Bq/m^3^ Exp.5: 180 Bq/m^3^ for 6 h	Exp.3: directly Exp.4: 4 h Exp.5: 24 h	Alcohol-induced oxidative damage; CCl_4_-induced hepathopathy	SOD activity Catalase activity ALD-activity and t-GSH in brain and liver	Protective effect of radon on oxidative damage	[126]
Kataoka et al., 2011	Mice (BALB/c, male, 7 weeks, 25 g)	*n*= 4–6	Inhalation, 18 kBq/m^3^ for 6 h	24 h	CCl_4_-induced hepatic and renal damage	t-GSH content, lipid peroxide levels, and GPx and GR activity in liver and kidney GOT, GPT, ALP activity, CRE, and T-CHO in serum	Radon inhalation inhibits oxidative damage of liver and kidney	[125]
Kataoka et al., 2011	Mice (BALB/c, male, 7 weeks, 25 g)	*n* = 5	Inhalation, 250, 500, 1000, 2000, or 4000 Bq/m^3^ for 0.5, 1, 2, 4, or 8 days	Directly	Healthy	SOD activity in brain, lung, thymus, heart, liver, stomach, pancreas, kidney	Activation of SOD; in plasma, brain, and lung strong and rapid response (enhancement); in liver, heart, pancreas, and small intestine only after low and high concentrations; in thymus and kidney after low concentration; no change in stomach	[129]
Kataoka et al., 2012	Mice (ICR, female, 8 weeks, 28 g)	*n* = 5–8	Inhalation, 1000 or 2000 Bq/m^3^ for 24 h or (L(+)-ascorbic acid injection or DL-α- tocopherol injection	24 h	CCl_4-_induced hepathopathy	SOD activity, catalase activity, GPx activity, t-GSH, LP levels and TG in the liver; GOT, GPT activity, TG and T-CHO levels in the serum; and histological examination of liver tissue	Decreased activities of GOT and GPT in serum; decreased TG levels in liver significantly higher SOD, catalase and GPx activity in livers; radon inhalation has an antioxidative effect against CCl4-induced hepatopathy that is comparable to treatment with AA or α-tocopherol	[127]
Kataoka et al., 2012	Mice (ICR, female, 8 weeks, 28 g)	*n* = 5–8	Inhalation, 1000 or 2000 Bq/m^3^ for 24 h or DL-α-tocopherol injection different concentrations)	24 h	CCl_4_-induced hepathopathy	SOD, catalase, t-GSH, and LP in kidneys CRE level in serum,	Decrease of CRE an LP levels; radon inhalation has an antioxidative effect comparable to the treatment with α-tocopherol at a dose of 300–500 mg/kg weight	[133]
Kataoka et al., 2012	Mice (ICR, female, 8 weeks, 28 g)	*n* = 6–7	Inhalation, 2000 Bq/m^3^ for 24 h	2 h	Carrageenan-induced inflammatory paw edema	SOD activity, catalase activity, t-GSH content, LP levels, TNF-α, NO, and paw histology.	Paw volume significantly decreased; lower TNF- α and NO levels; SOD activity increased; fewer infiltrating leukocytes; increased SOD and catalase activities	[134]
Nishiyama et al., 2012	Mice (BALB/c, male, 7 weeks, 23 g)	*n* = 8	Inhalation, 2000 Bq/m^3^ for 8 days	Directly	Dextran sulfate sodium (DSS) model of colitis (while radon exposure)	MPO, NO, TNF-α, SOD, CAT, t-GSH), LPO level, and Histology, DAI and weight gain	Significant lower DAI score; less shortened colon; lower plasma TNF- α and MPO activity in colon; enhanced SOD activity and tGSH content; lower LPO level in the colon and NO level in plasma	[135]
Toyota et al., 2012	Mice (C57BL/6J, male, 8 weeks, 20 g)	*n* = 4–6	Inhalation, 4000 Bq/m^3^ for 24 h	6 and 24 h	Acute alcohol-induced hepatopathy	SOD, catalase, t-GSH, GPx, GR, TG, and lipid peroxide in liver, GOT and GPT, activity and the TG, T-CHO in serum	Radon treatment activates antioxidative functions and inhibits acute alcohol-induced oxidative damage, hepatopathy and fatty liver in mice	[136]
Nishiyama et al., 2013	Mice, (C57BL/6J, male, 9 weeks, 25–28 g)	*n* = 5–8	Inhalation, 1000, 2500, and 5500 Bq/m3 for 24 h	4 days	Streptozotocin-induced Type-1 Diabetes (after radon exposure)	SOD activity, CAT activity, t-GSH content, LPO, blood glucose, serum insulin, and body weight	Higher SOD activity and t-GSH content, lower LPO levels; significantly suppressed blood glucose elevation and body weight decrease; higher serum insulin; radon inhalation partially suppressed type-1 diabetes induced by STZ administration	[137]
Yamato et al., 2013	Mice (male ICR, 8 weeks, 38 g)	*n* = 5–10	Inhalation, 1000 or 2000 Bq/m^3^ for 24 h	Up to 35 min (licking response), no information for other endpoints	Formalin-induced transient inflammatory pain	licking response (pain), TNF-α, NO, paw histology, SOD and CAT activities, total glutathione (t-GSH) content, and LPO levels	Enhanced SOD-activity, t-GSH content in serum and paws, reduced number of leukocytes, reduced TNF-α and NO level	[138]
Etani et al., 2016	Mice (male, 8 weeks, 32–38 g)	*n* = 8–9 (drinking treatment) *n* = 6 (inhalation)	Drinking water: 338 ± 11 Bq/L for 2 weeks Inhalation: 2000 Bq/m^3^ for 24 h	3 h	PO model of hyperuricemia (induced after radon treatment)	Activities of XOD, SOD andCAT; levels of t-GSH and proteins in liver and kidney	Radon-inhalation activates antioxidative function and reduces serum uric acid levels	[139]
Kataoka et al., 2016	Mice (ICR, male, 8 weeks; 33–40 g)	*n* = 5–6	Inhalation, 1000 Bq/m^3^ for 24 h and/or pregabalin treatment.	30 min, 60 min, 90 min, 120 min	CCI—induced neuropathic pain	von Frey Test (pain), SOD activity, catalase activity, t-GSH content, and LP level in paw.	Pregabalin and radon has mitigative effect on pain after CCI due to antioxidative function after radon inhalation	[140]
Etani et al., 2017	Mice (BALB/c, male, 8 weeks, 25–28 g)	*n* = 8 (drinking treatment) *n* = 8 (inhalation)	Drinking water: 663 ± 36 Bq/L for 2 weeks Inhalation: 2000 Bq/m^3^ for 24 h	1 h	Gastric mucosal injury induced by oral ethanol administration (induced after radon treatment)	UI and HI: SOD and CAT activity, and the levels of t-GSH in stomachs	Lower UI and IHI after radon treatment; activation of antioxidative mechanisms	[141]
Kataoka et al., 2017	Mice (BALB/c, male, 8 weeks, 24–28 g)	*n* = 7	Inhalation, 500–2000 Bq/m^3^ for 24 h	Unclear	Healthy	NF-κB, NIK, IKK-β, ATM; total SOD, Mn-SOD and Cu/Zn-SOD activities and protein levels	Induction of SOD proteins, mainly Mn-SOD; Mn-SOD induced by NF-κB activation stimulated by DNA damage and oxidative stress	[130]
Pei et al., 2017	Mice, (BALB/c, male, 15 g)	*n* = 6	Inhalation, 100,000 Bq/m^3^, 12 h/d, for up to cumulative doses of 60 WLM	Directly	Healthy	circRNA, H&E, Caspase 3	Enhanced Caspase 3 expression, circRNA profiles are changed	[142]
Paletta et al. 1975	Rat (male, 200 g)	*n* = 5	Series 1: Rn 12.5 nCi/L, RaB/Rn 0,25; Series 2: Rn 110 nCi/L, RaB/Rn 0,33 Different doses to organs?	12 d	Healthy	Corticosteroid level in serum	2 maxima of corticosteroid after exposure, one after 8 h, one after 5 (low) or 9 h (high concentration)	[143]
Taya et al., 1994	Rat (male, 4–6 months old)	*n* = 10–25	120–990 WLM (dose rate 7–9 WLM/h; 725–770 Bq/m3)	7–28 d	Healthy	Proliferation in epithelial cells of respiratory tract; binucleate alveolar macrophages (AM) and/or micronuclei	Labelling indices increased after exposure; highest in bronchial epithelial cells; binculeate AM as well as induction of micronuclei was increased after exposure; binucleate AM with micronuclei were only induced in exposed animals; no inflammation	[144]
Ma et al., 1996	Rats (Wistar, male, 30 weeks)	*n* = 3	Inhalation, 1000–5000 kBq/m^3^ or 400–1600 kBq/m^3^ for 4 or 16 h	Directly	Healthy	SOD activity in blood, kidney, spleen, and liver	Increase after 4 h, decrease after 16 h of exposure	[128]
Collier et al., 1997	Rats (Sprague-Dawley, male, 2–12 month,	*n* = 2–6	Inhalation, 200–1600 WLM, 250–7142 WL for 1–27.5 days	14 d	Healthy	Cell number, nuclear abberations, number of macrophages and macrophage proliferation in lung lavage fluid, H&E and BrdU staining of lung sections	Positive dose-response for most effects	[145]
Cui et al., 2008	Rats (Wistar)	*n* = 6	Inhalation; 60, 90, and 120 working level months (WLM) in total; inhalation for 8 h per day, 6 days per week	No information	Healthy	MNR, hprt assay in lymphocytes, and tracheal-bronchial epithelial cells	Dose-dependent increase of MNR, the mutation frequency of hprt is increased with accumulated dose, can be used as biomarkers for genetic changes after radon exposure	[146]
Yamaoka et al., 1993	Rabbits	*n* = 10–14	Inhalation of nebulized radon water; 7–10 kBq/L or 14–18 kBq/L	Directly and 2 h	Healthy	Lipid peroxide, SOD, membrane fluidity in brain, spleen, lung, liver and serum	Enhanced SOD activity, reduced lipid peroxide levels	[147]
Kataoka et al., 2014	Mongolian gerbil MGS/sea, (female, 8 weeks, 50 g)	*n* = 5–7	Inhalation, 2000 Bq/m^3^ for 24 h	Directly	Transient global cerebral ischemia induced by bilateral occlusion of the common carotid artery (3 days before radon treatment)	Brain histology, SOD activity, CAT activity, and t-GSH content in the brain and serum.	Number of damaged neurons significantly lower; increased SOD activity; unchanged t-GSH	[148]

Abbreviations: WT: wild type, PO: potassium oxonate, UI: ulcer index, IHI: index of histological injury, SOD: superoxide dismutase, XOD: xanthine oxidase, CAT: catalase, GPx: glutathione peroxidase, GR: glutathione reductase, GOT: glutamic oxaloacetic transaminase, GPT: glutamic pyruvic transaminase, ALP: alkaline phosphatase, CRE: creatinine, T-CHO: total cholesterol, LP: lipid peroxidase TG: triglyceride, AA: L(+)-ascorbic acid, TNF-α: tumor necrosis factor alpha, t-GSH: total glutathione content, NO: nitric monoxide, CCI: chronic constriction injury, NIK: NF-κB–inducing kinase, IKK-β: inhibitor of κB kinase-β, ATM: ataxia-telangiectasia mutated kinase, MPO: myeloperoxidase, DAI: disease activity index, WLM: working level months, hprt hypoxanthine phosphoribosyl transferase, MNR: micronuclei rate.

## Data Availability

Not applicable.

## Appendix A

**Table A1:** Some of today’s radon spas all over the World [87,88,90,162].

Country	Place (City)
Austria	Bad Gastein, Bad Hofgastein, Bad Zell, Gasteiner Heilstollen
Bulgaria	Hisarja
Czech Republic	Jáchymov
Chile	Jahucl Hot Springs
China	Nanshui, Taishan
France	Plombiers
Germany	Bad Brambach, Bad Kreuznach, Bad Münster am Stein, Bad Schlema, Bad Steben, Sibyllenbad, Menzenschwand St. Blasien, Weissenstadt
Greece	Ikaria, Polichnitos, Eftalou
Hungary	Abaliget Cave, Budapest, Beke Cave, Eger, lstván Cave, Tapolca Hospital Cave, Szemlöhegy Cave
Italy	Ischia, Meran
Japan	Misasa
Poland	Długopole-Zdrój, Ladek-Zdrój, Świeradów-Zdrój, Szczawno-Zdrój, Przerzeczyn-Zdrój
Romania	Felix Spa
Russia	Pyatigorsk (Caucasus). Belokuriha (Altai, Siberia) and Yangan Tau (Ural)
Ukraine	Khmelnik
USA	Boulder (Montana)
